# Detection of Avian Orthoavulavirus-1 genotypes VI.2.1 and VII.1.1 with neuro-viscerotropic tropism in some backyard pigeons (Columbidae) in Eastern Saudi Arabia

**DOI:** 10.3389/fvets.2024.1352636

**Published:** 2024-02-27

**Authors:** Abdullah I. A. Al-Mubarak, Anwar A. G. Al-Kubati, Abdullah Sheikh, Adel M. Abdelaziz, Jamal Hussen, Mahmoud Kandeel, Baraa Falemban, Maged Gomaa Hemida

**Affiliations:** ^1^Department of Microbiology, College of Veterinary Medicine, King Faisal University, Al-Hofuf, Saudi Arabia; ^2^Department of Veterinary Medicine, Faculty of Agriculture and Veterinary Medicine, Thamar University, Dhamar, Yemen; ^3^Camel Research Center, King Faisal University, Al Hofuf, Saudi Arabia; ^4^Faculty of Veterinary Medicine, Veterinary Educational Hospital, Zagazig University, Zagazig, Egypt; ^5^Veterinary Diagnostic Laboratory, Ministry of Environment, Water and Agriculture, Al-Ahsa, Saudi Arabia.; ^6^Department of Biomedical Sciences, College of Veterinary Medicine, King Faisal University, Al-Hofuf, Saudi Arabia; ^7^Department of Pharmacology, Faculty of Veterinary Medicine, Kafrelsheikh University, Kafrelsheikh, Egypt; ^8^Department of Veterinary Biomedical Sciences, College of Veterinary Medicine, Long Island University, Brookville, NY, United States

**Keywords:** Avian Orthoavulavirus-1 (AOAV1), pigeons, natural infection, RT-PCR, genotype VI, genotype VII, neurotropic, viscerotropic

## Abstract

**Introduction:**

Avian orthoavulavirus-1 (AOAV1) has a wide host range, including domestic and wild birds. The present study aimed to identify the currently circulating AOAV1 strains from some outbreaks in some backyard pigeons in the eastern region of Saudi Arabia (ERSA).

**Methods:**

Tracheal/cloacal swabs and tissue specimens were collected from eight backyards in Al-Ahsa, ERSA, between January 2021 and March 2023. Samples were tested for the presence of AOAV1 using commercial real-time RT-PCR. Part of the fusion gene was also amplified by gel-based RT-PCR, and the obtained amplicons were sequenced.

**Results and discussion:**

AOAV1 was detected in samples from the eight flocks. The retrieved sequences from samples of 6/8 pigeon backyards are reported. Phylogenetic analysis based on the obtained sequences from these backyard pigeons showed the segregation of the obtained sequences in AOAV1 genotypes VI.2.1 and VII.1.1. Clinically, nervous manifestations were dominant in pigeons infected with both genotypes. Respiratory manifestations and significantly higher overall mortality rate were induced by genotype VI.2.1. The deduced amino acid sequences of the fusion protein cleavage site (FPCS) showed that all the detected isolates belong to velogenic strains. Differences in clinical profiles induced by the natural infection of pigeons with AOAV1 genotypes VI.2.1 and VII.1.1 were reported. The present findings highlight the potential roles of some backyard pigeons in the long-distance spread and cross-species transmission of the reported AOAVI genotypes. Further research is required to perform biotyping and pathotyping of the reported strains.

## 1 Introduction

Avian Orthoavulavirus-1 (AOAV1), formerly termed Avian Paramyxovirus 1 (APMV1), belongs to the genus *Orthoavulavirus* of the subfamily *Avulavirinae*, family *Paramyxoviridae*. AOAV1 possesses a single-stranded negative-sense RNA genome of about 15 kb in length that encodes six genes (3′-N-P-M-F-HN-L-5′) (1). AOAV1 can be classified according to their sequence similarity (genotyping), according to their ability to induce death and/or nervous signs in inoculated embryonated eggs or chicks [mean death time (MDT) and intracerebral pathogenicity index (ICPI)], and according to the clinical signs they induce in naturally infected chickens. The fourth gene, Fusion (F), is frequently targeted by molecular diagnostic techniques for detection, sequencing, and genotyping of the AOAV1. Based on complete F gene sequences, AOAV1 has been classified into two major classes: I and II. Class II includes virulent and avirulent viruses, and it is subclassified into 21 genotypes (I to XXI) and many subgenotypes (2). AOAV1 may also be classified according to the score of the ICPI into lentogenic (< 0.7), mesogenic (0.7-1.5), and velogenic (>1.5) strains (3). Alternatively, the World Organization of Animal Health (WOAH) recommended the use of the deduced amino acid sequence of the F protein cleavage site (FPCS) as a non-invasive technique to assess the virulence of the AOAV1 isolates (4). Based on the clinical signs, AOAV1 infection in chickens was classified into five types: asymptomatic enteric, lentogenic, mesogenic, velogenic viscerotropic, and velogenic neurotropic (4). Differentiation between velogenic viscerotropic and velogenic neurotropic strains demands evaluation of the clinical disease or an intravenous test in adult birds (3).

AOAV1 affects a wide range of bird species, such as chickens, turkeys, pigeons, and wild bird species (2, 5). This virus induces considerable economic losses to the poultry industry worldwide (6). Additionally, AOAV1 can affect humans and cause mild, transient conjunctivitis, though rare, severe infections were also reported in immunocompromised patients (7, 8). The virus remains endemic in a wide part of the world, and some of the affected birds were implicated in the maintenance of the virus (9). Virulent AOAV1 was reported in migratory waterfowl (10). In fact, it has been demonstrated that similar AOAV1 strains are circulating simultaneously in both domestic and wild birds (11–13), suggesting a potential role in the dissemination of virulent AOAV1 (5, 14, 15). The role of some free-ranging birds, like pigeons, in the transmission of virulent NDV to poultry is also a major concern (16–18).

AOAV1 has been reported in Saudi Arabia in the 1980s (19). The AOAV1 genotypes II, VI, VII, XII, XIII, XX, and XXI have been reported in Saudi Arabia and many surrounding countries (2, 20–23). The diversity of the reported genotypes implies the high vulnerability of this region to the spread of AOAV1 and dictates continuous monitoring of this virus. Limited data is available on the circulating AOAV1 genotypes in non-chicken flocks, such as pigeons, in Saudi Arabia. Pigeon flocks in Al-Ahsa, ERSA, have experienced disease with manifestations suggestive of AOAV1 infection during the years 2021 and 2022. Swabs and tissue samples were collected from affected flocks; nucleic acid was extracted from the collected samples and used in an RT-PCR assay to detect AOAV1. The F-gene was also targeted for sequencing and genotyping of the detected AOAV1. Herein, we report on findings from disease investigation, detection, and genotyping of AOAV1 in some affected pigeon flocks in ERSA.

## 2 Materials and methods

### 2.1 Ethical approval

All management conditions and experimental procedures used in the present study were approved by the Ethics Committee at King Faisal University, Saudi Arabia (KFU-REC-2022-MAR-EA000541). Additionally, the experimental protocols and all management conditions in the study were conducted following the regulations, husbandry, ethics, and approved guidelines of the Institutional Animal Care and Use Committee of the Faculty of Veterinary Medicine, Zagazig University, Egypt under the reference number of ZU-IACUC/2/F/444/2023.

### 2.2 Sample collection and processing

The study was conducted in Al-Ahsa governorate in the ERSA during the period from January 2021 to March 2023. Pigeons flocks showing signs suggestive of AOAV1 infection were targeted in the present study. Epidemiological and clinical data were reported. The birds showing nervous manifestations were euthanized by cervical dislocation following standard procedures according to Anon (24). A diagnostic postmortem inspection was conducted, and the main lesions were systematically reported. A total of 64 cloacal/tracheal swabs and 147 tissue/organ specimens were collected from eight backyard pigeon flocks in ERSA at this time window. Tracheal and cloacal swabs were collected on viral transport media prepared from Dulbecco's Modified Eagle's Medium, and antibiotics (2,000 U/ml penicillin, 2 mg/ml streptomycin, 50 μg/ml gentamycin), anti-mycotic (50 U/ml nystatin) and bovine serum albumin (0.5%) (Sigma-Aldrich, USA). Swabs were thoroughly vortexed for 2 min and then stored at (−80°C). Ten percent of tissue homogenates were prepared from collected tissue samples (trachea, lung, glandular stomach, spleen, liver, and brain) using silica beads and Tissue-Lyser (Qiagen, Hilden, Germany). The obtained supernatants were then stored at (−80°C) for further analysis.

### 2.3 Detection of AOAV1 by real-time RT-PCR

Viral RNA was extracted from the processed swabs and tissue suspensions using the MagNA Pure Compact Nucleic Acid Isolation Kit (Cat # 03 730 964 001, Roche Diagnostic Gmbh, Mannheim, Germany) according to the manufacturer's instructions. Extracted viral RNAs were stored at (−20°C) until used in a subsequent molecular procedure. For the detection of the AOAV1 nucleic acid, the commercially-available real time RT-PCR reagents developed and manufactured by TIB MOLBIOL Gmbh berline, Germany were used. Forward and reverse primers and probes used according to the method of Wise et al. (25) and produced by TIB MOLBIOL Gmbh berline, Germany. Briefly, the provided specific primers were used to amplify a 99-bp fragment of the AOAV1-fusion gene, and the amplified fragment was detected using a hybridization probe labeled with Light Cycler Red 640. The test was considered valid only if the negative control showed no signal, the low concentration NDV DNA samples should show an amplification curve for IC with Cp at approximately cycle 28–30. The kit provides standard row of clone and purified DNA with concentration in the range from 105 to 101 copies/rxn of NDV should have Cp value (19–23, 26–38).

### 2.4 Genotyping of the detected AOAV1 amplicons

According to the manufacturer's instructions, the Reverse Transcription System (Cat # A3500, Promega, USA) was used to reverse transcribe of the extracted viral RNA. The obtained cDNA was then stored at (−80°C) until used in a PCR test. The previously described primers (Table 1) and thermal protocols were used to amplify the fusion gene of the AOAV1 (27, 28). The PCR assay was performed using the Go-Taq Green Master Mix (Cat #M7122, Promega, USA) in a final volume of 25 μl according to the manufacturer's procedure. Thermal manipulation was performed using the Bio-Rad T-100 thermal cycler. PCR products were separated by electrophoresis in a 1% agarose gel in Tris-borate-EDTA buffer with red-safe nucleic acid staining dye (Cat #21141, iNtRON, Biotechnology, Burlington, USA). Targeted bands of correct size were excised, and amplicons were purified using the Wizard SV Gel and PCR Clean-up System (Cat # A1460, Promega) according to the manufacturer's instructions. Purified amplicons were sequenced at Macrogen Sequencing Service, South Korea, using the same primers as for the PCR.

**Table 1:** The primers used in the genotyping RT-PCR in the present study.

**Name**	**Primers sequence**	**Product length (nt)**	
F primers	NDV EXT-F	GCAGCTGCAGGGATTGTGGT	356	(27)
	NDV EXT-R	TCTTTGAGCAGGAGGATGTTG		
I primers	NDV INT-F	CCCCGTTGGAGGCATAC	216	
	NDV INT-R	TGTTGGCAGCATTTTGATTG		
N primers	NDVU	GGAGGATGTTGGCAGCATT	310	(28)
	NDVD	GTCAACATATAC ACCTCATC		

For both PCR assays, internal extraction control were used in parallel to the tested samples to evaluate the RNA extraction process. Additionally, positive internal control were used for evaluation of amplification of target gene as well as negative control to exclude cross contamination. The negative control was setup with all the ingredients except the cDNA which was replaced with nuclease free water.

### 2.5 Sequence analysis and phylogenetic analysis

Sequences were handled using the Molecular Evolutionary Genetics Analysis (MEGA) 11 software (29). Sequences were aligned using the ClustalW method with default setting. Genbank BLAST search was used to retrieve most similar sequences to those reported in the present study. Sequences with identity of ≥95% were involved in the analysis after removal of those showing high identity to each other (≥99). Sequences from Saudi Arabia and gulf region were also involved in the analysis. The phylogenetic analysis was performed according to the genotyping and nomenclature system of the AOAV1 developed by (2). Phylogenetic trees were prepared using the Neighbor-Joining method in MEGA11 with a bootstrap value of 1,000 replicates. Sequence identity was calculated by dividing the number of matched nucleotides by the total length of the aligned pairs of sequences.

### 2.6 Data analysis

Data was handled using MS Excel. The Fisher's exact test was used to reveal the significance of the differences in morbidity and mortality rates induced by detected genotypes. Significance was defined as a *p* < 0.05.

### 2.7 Genbank accession number (GB#)

The reported pigeon-AOAV1 sequences in the present study were deposited in the Genbank under the GB# shown in Table 4.

## 3 Results

### 3.1 Molecular detection and genotyping

AOAV1 was detected by real-time RT-PCR in the samples collected from the investigated eight pigeon flocks. Regarding the genotyping RT-PCR, partial F gene sequences were successfully recovered from samples of 4/8 pigeon flocks (F1 to F4) based on the F-primers (Table 1). Subsequently, the I and N primers were also used in attempts to obtain sequences from samples of the remaining pigeon flocks. Sequences were recovered from samples of two additional pigeon flocks using primers I (I2 and I3).

**Figure 1:**
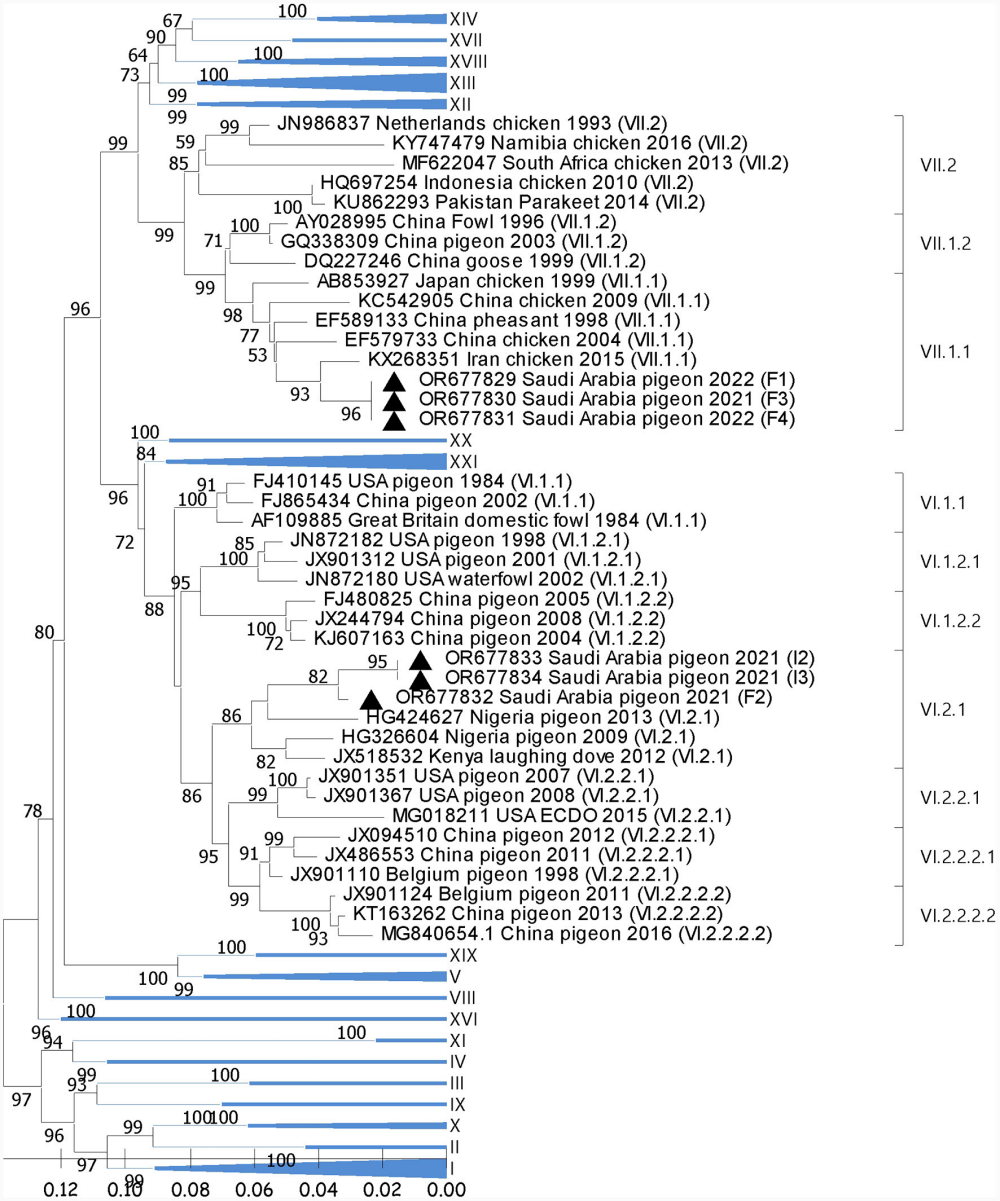
Phylogenetic analysis of the reported AOAV1 sequences from pigeons. The tree was constructed using the NJ alignment with a bootstrap value (=1,000). The black triangle indicates the present detections of AOAV1. GB# of sequences are shown, followed by country, host, and collection year of the sequenced samples. Genotypes of the published sequences are shown in brackets.

Phylogenetic analysis showed the segregation of 3/6 sequences (F1, F3, and F4) with AOAV1-subgenotype VII.1.1 (VII. l). These sequences showed an internal identity of 100%. The most similar (97.2%) published sequence (GB# KX268351) was recovered from chickens in Iran in 2015 (Figure 1). The remaining three sequences (F2, I2, and I3) showed an overall similarity of 97.8%-100% and segregated with subgenotype VI.2.1. These sequences clustered with the reference sequence (GB# JX518532) that was recovered from a laughing dove in Kenya in 2012 with (95.1%−95.2%) similarities. Additionally, it showed 94.1% to 95.4% similarity with the published sequence (GB#HG326604) that was recovered from pigeons in Nigeria in 2009. The sequence identity of the two groups with published sequences of the AOAV1 genotypes and subgenotypes is shown in Table 2.

**Table 2:** Averaged identity of recovered AOAV1 sequences of each genotype with reference sequences and most similar local and regional sequences according to BLAST search.

**Genotype VI**				**Genotype VII**			
GB#_Country_host_year of collection (genotype for reference sequences)	F2^*^	I2	I3	GB#_Country_host_year of collection (genotype for reference sequences)	F1	F3	F4
HG424627_Nigeria_pigeon_2013_(VI.2.1)	94.8	94.1	94.1	KX268351_Iran_chicken_2015_(VII.1.1)	97.2	97.2	97.2
HG326604_Nigeria_pigeon_2009_(VI.2.1)	95.4	94.1	94.1	EF589133_China_pheasant_1998_(VII.1.1)	96.6	96.6	96.6
JX518532_Kenya_laughing dove_2012_(VI.2.1)	95.1	95.2	95.2	AB853927_Japan_chicken_1999_(VII.1.1)	96	96	96
JX486553_China_pigeon_2011_(VI.2.2.2.1)	93.2	92.5	92.5	EF579733_China_chicken_2004_(VII.1.1)	95.7	95.7	95.7
FJ865434_China_pigeon_2002_(VI.1.1)	94.1	93	93	KC542905_China_chicken_2009_(VII.1.1)	94.8	94.8	94.8
JX901351_USA_pigeon_2007_(VI.2.2.1)	92.6	93	93	DQ227246_China_goose_1999_(VII.1.2)	94.1	94.1	94.1
FJ410145_USA_pigeon_1984_(VI.1.1)	93.8	92.5	92.5	GQ338309_China_pigeon_2003_(VII.1.2)	94.1	94.1	94.1
JX901110_Belgium_pigeon_1998_(VI.2.2.2.1)	94.4	93.6	93.6	AY028995_China_Fowl_1996_(VII.1.2)	93.8	93.8	93.8
JN872180_USA_waterfowl_2002_(VI.1.2.1)	93.5	91.4	91.4	KY747479_Namibia_chicken_2016_(VII.2)	90.5	90.5	90.5
JX901367_USA_pigeon_2008_(VI.2.2.1)	92.9	93	93	KU862293_Pakistan_Parakeet_2014_(VII.2)	92	92	92
JX901124_Belgium_pigeon_2011_(VI.2.2.2.2)	93.2	92.5	92.5	MF622047_South Africa_chicken_2013_(VII.2)	90.8	90.8	90.8
KT163262_China_pigeon_2013_(VI.2.2.2.2)	93.2	92.5	92.5	HQ697254_Indonesia_chicken_2010_(VII.2)	92	92	92
JX901312_USA_pigeon_2001_(VI.1.2.1)	93.2	91.4	91.4	JN986837_Netherlands_chicken_1993_(VII.2)	94.1	94.1	94.1
AF109885_Great Britain_domestic fowl_1984_(VI.1.1)	93.2	92	92	MZ747024_Iran_chicken_2021	98.4	98.4	98.4
JN872182_USA_pigeon_1998_(VI.1.2.1)	92.6	91.4	91.4	MN481201_Iran_chicken_2019	98.2	98.2	98.2
JX094510_China_pigeon_2012_(VI.2.2.2.1)	92	92	92	MW295940_Pakistan_Chicken_2019	98.2	98.2	98.2
KJ607163_China_pigeon_2004_(VI.1.2.2)	92	90.9	90.9	MH247187_Iran_chicken_2017	98.5	98.5	98.5
JX244794_China_pigeon_2008_(VI.1.2.2)	91.4	90.4	90.4	MK659697_Iran_chicken_2017	98.5	98.5	98.5
MG018211_USA_ECDO_2015_(VI.2.2.1)	90.5	91.4	91.4	MK659700_Iran_Eurasian_magpie_2017	98.5	98.5	98.5
MG840654_China_pigeon_2016_(VI.2.2.2.2)	91.4	90.4	90.4	MN481193_Iran_chicken_2018	98.5	98.5	98.5
FJ480825_China_pigeon_2005_(VI.1.2.2)	90.5	89.3	89.3	MH247184_Iran_chicken_2017	98.2	98.2	98.2
KR014202_Ethiopia_Pigeon_2014	95.9	95.3	95.3	MK659698_Iran_chicken_2017	98.2	98.2	98.2
HQ456844_Nigeria_chicken_2008	95.8	94.3	94.3	OK338510_Malaysia_Chicken_2021	97.6	97.6	97.6
JQ039387_Nigeria_pigeon_2008	95.7	94.7	94.7	OK338511_Malaysia_Chicken_2021	97.6	97.6	97.6
JQ039388_Nigeria_pigeon_2008	95.7	94.7	94.7	OK338512_Malaysia_Chicken_2021	97.6	97.6	97.6
JQ039391_Nigeria_pigeon_2007	95.7	94.7	94.7	OK338513_Malaysia_Chicken_2021	97.6	97.6	97.6
KY292310_Nigeria_quail_2009	95.7	94.7	94.7	OK338514_Malaysia_Chicken_2021	97.6	97.6	97.6
MH996920_Nigeria_quail_2008	95.7	94.7	94.7	OK338515_Malaysia_Chicken_2021	97.6	97.6	97.6
HG326601_Nigeria_pigeon_2007	96	95.2	95.2	MT370496_Iraq_Chicken_2008	96.6	96.6	96.6
HG326602_Nigeria_pigeon_2007	96	95.2	95.2	MT370499_Iraq_Chicken_2005	96.9	96.9	96.9
HG326603_Nigeria_pigeon_2007	95.7	95.2	95.2	MH377249_Israel_peacock_2008	96	96	96
MH996992_Nigeria_pigeon_2015	95.4	94.7	94.7	GU289472_China_chicken_2006	95.7	95.7	95.7
FM200798_Nigeria_parrot_2007	95.7	94.7	94.7	KC851848_Ethiopia_chicken_2012	95.8	95.8	95.8
FM200797_Nigeria_pigeon_2007	95.4	94.1	94.1	GU289468_China_chicken_2006	95.3	95.3	95.3
OR122679_Saudi_Arabia_chicken_2022	99.4	97.9	97.9	OR122680_Saudi_Arabia_chicken_2022	99.7	99.7	99.7
AY471783_UAE_kestrel_1998	94.3	94.5	94.5	OR122682_Saudi_Arabia_chicken_2022	99.7	99.7	99.7
AY471784_UAE_kestrel_1998	94.3	94.5	94.5	OR122681_Saudi_Arabia_chicken_2022	99.4	99.4	99.4
AY471779_UAE_pigeon_1998	94.3	93.8	93.8	OR122683_Saudi_Arabia_peacock_2022	99.4	99.4	99.4
AY471781_UAE_pigeon_1996	94.3	94.5	94.5	MG022111_Saudi_Arabia_chicken_2015	96.6	96.6	96.6
AY471782_UAE_pigeon_1997	94.3	93.8	93.8	MG022113_Saudi_Arabia_chicken_2016	96.6	96.6	96.6
AY471785_UAE_kestrel_1999	94.3	94.5	94.5	MG022114_Saudi_Arabia_chicken_2016	96.6	96.6	96.6
AY471780_UAE_pigeon_1999	93.9	93.8	93.8	MG022113_Saudi_Arabia_chicken_2016	96.6	96.6	96.6
AY471786_Saudi_Arabia_pigeon_1998	93.9	92.2	92.2	MK608022_Saudi_Arabia_chicken_2013	93	93	93

### 3.2 Comparison of the similarity of the reported sequences with local and regional AOAV1 sequences

**Figure 2:**
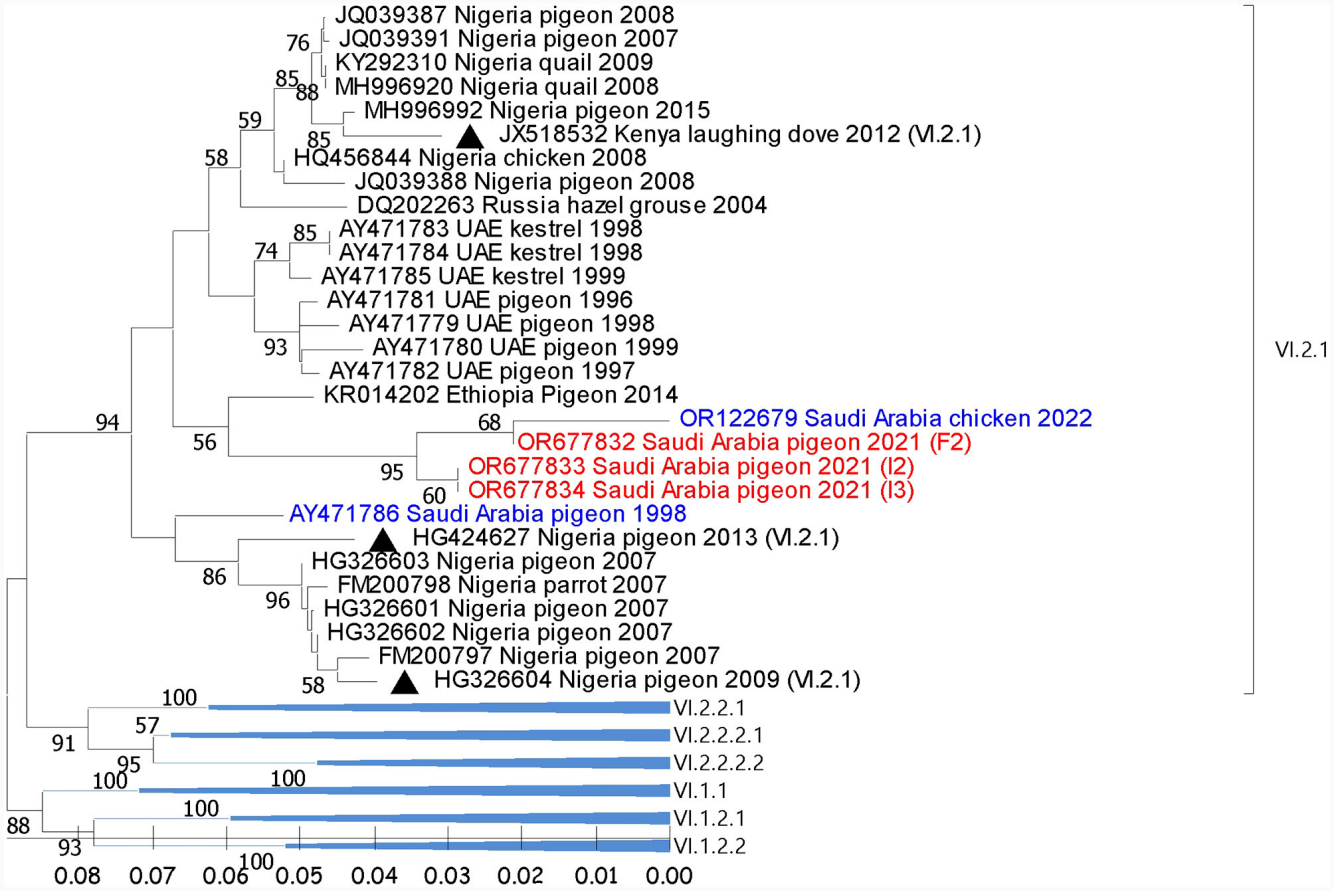
Phylogenetic analysis of the reported AOAV1-VI.2.1 sequences from pigeons and most similar sequences. The tree was constructed using the NJ alignment with a bootstrap value of 1,000. The black triangles indicate the reference sequence; the blue ones are the previous detections from Saudi Arabia; the red ones are the present detections of AOAV1. GB# of sequences are shown, followed by country, host, and collection year of the sequenced samples. Genotypes of the reference sequences are shown in brackets.

Reported sequences in the subgenotype VI.2.1 showed (97.9%-99.4%) similarity with the (GB# OR122679; SA/Chicken/NDV1/2022) sequence isolated from backyard chickens in Al-Ahsa in 2022. Regionally, these sequences showed (94.4%) similarity with (GB# AY471783), isolated from Kestrel in the UAE in 1998. It also showed (95.3%-95.9%) identity with the (GB# KR014202) sequence that was recovered from pigeons in Ethiopia in 2014 (Figure 2). The BLAST analysis showed that sequences with ≥95% similarity were mainly isolated from pigeons and rarely from quail, parrots, and laughing doves through the period from 2004 to 2019 from Nigeria, Kenya, Ethiopia, Iran, Iraq, Turkey, Russia, and China.

**Figure 3:**
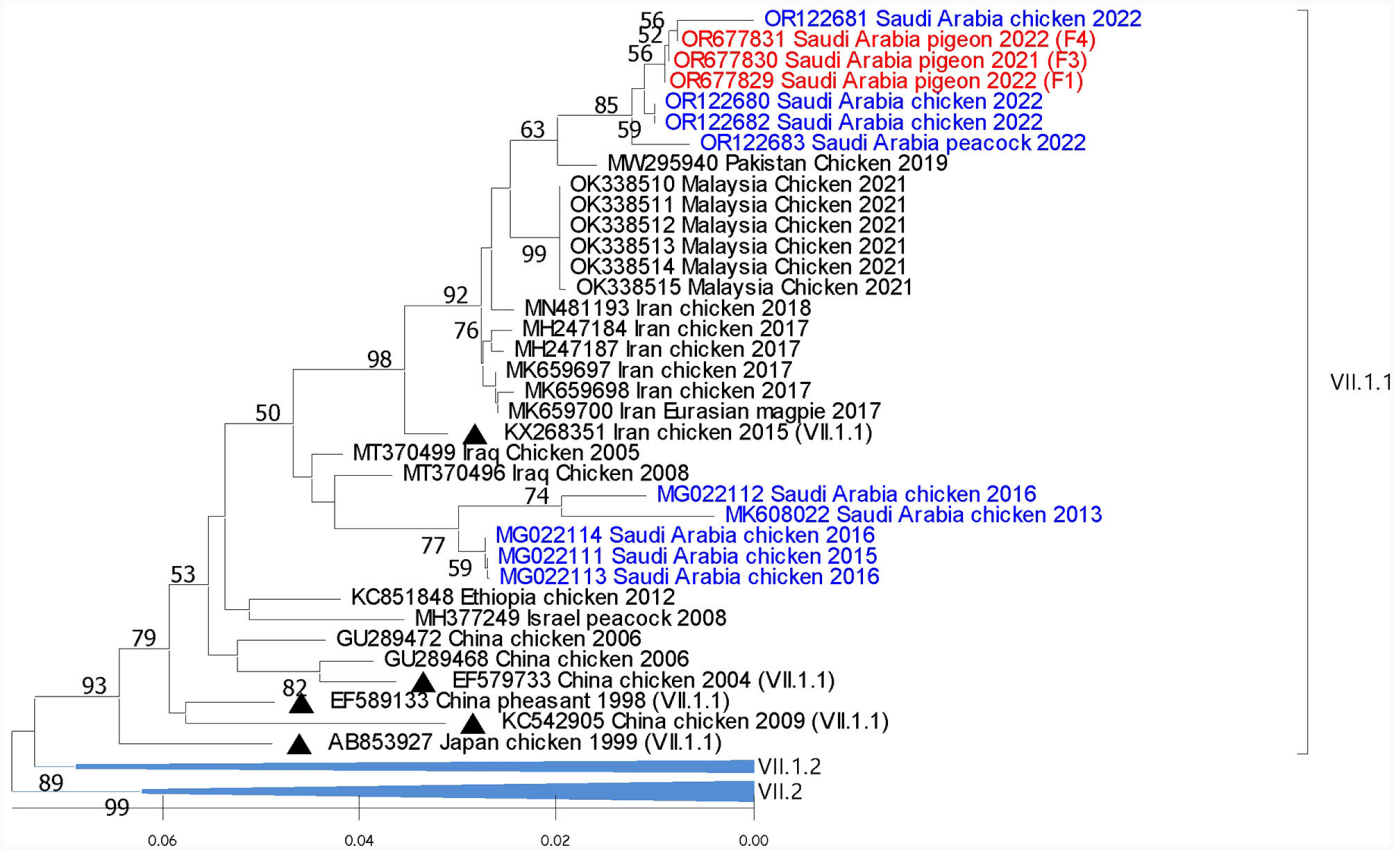
Phylogenetic analysis of the reported AOAV1-VII.1.1 sequences from pigeons and most similar sequences. The tree was constructed using the NJ alignment with a bootstrap value of 1,000. The black triangles indicate the reference sequence; the blue ones are the previous detections from Saudi Arabia, and the red ones are the present detections of AOAV1. GB# of sequences are shown, followed by country, host, and collection year of the sequenced samples. The genotypes of the reference sequences are shown in brackets.

Reported sequences in the subgenotype VII.1.1 showed 99.4% to 99.7% similarity with sequences recovered from chickens and peacocks in Al-Ahsa in 2022. A lower (93% to 96.6%) similarity was shared with sequences that were isolated from chickens in Saudi Arabia in 2013 to 2015. Regionally, these sequences showed (98.4%) similarity with the (GB# MZ747024) sequence that was isolated from chickens in Iran in 2021 (Figure 3).

### 3.3 Epidemiological and clinical data of the investigated pigeon flocks

**Figure 4:**
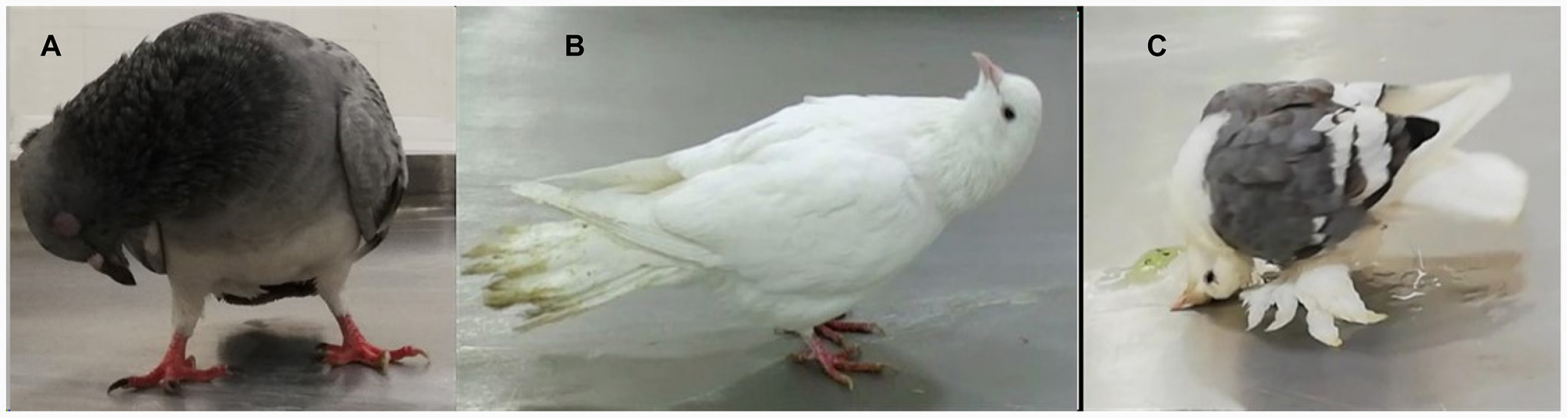
Nervous signs (head and neck twist) **(A–C)** and greenish diarrhea **(B, C)** with a soiled tail feather **(B)** in pigeons infected with AOAV1 in the present study.

Infected pigeon flocks showed morbidity rates ranging between 25% and 50%, while mortality rates ranged between 8.3% and 21.3% (Table 3). Some pigeons or squabs were found dead without any clinical signs. In others, the disease started with depression and loss of appetite, mild respiratory signs, and greenish diarrhea. These were followed by the appearance of nervous manifestations that commence with wing and leg paralysis, recumbency, or inability to walk and fly. In some cases, severe nervous signs follow and include circling, loss of balance, torticollis, neck paralysis, or twisting (Figure 4), and then finally death. Necropsy of recently died pigeons or euthanized diseased pigeons revealed the presence of congestion/hemorrhages in the trachea, lungs, liver, spleen, and brain. Petechial hemorrhages were also observed on proventriculus and cecal tonsils.

**Table 3:** Epidemiological, clinical, and postmortem data of the sampled pigeon flocks.

**Sample/ sequence ID**	**Date of collection**	**Location**	**Flock size**	**Morbidity (%)**	**Mortality (%)**	**Clinical signs and post mortem lesions**	**No of swab samples1**	**No of tissue samples2**
SA/Pigeon/I3/2021	Jan. 2021	A	300	82 (27.33)	52 (17.33)	Mild respiratory signs, nervous signs, brain congestion, and petechial hemorrhages in proventriculous	10	20
SA/Pigeon/F2/2021	March 2021	B	150	57 (38)	32 (21.33)	Respiratory signs, paralysis, brain congestion, and green diarrhea	8	16
SA/Pigeon/F3/2021	Sept. 2021	F	180	45 (25)	15 (8.33)	Wing paralysis, pneumonia, liver congestion, and air saculitis	10	25
SA/Pigeon/I2/2021	Dec. 2021	D	55	17 (30.91)	11 (20)	Respiratory signs, nervous signs, paralysis, and greenish diarrhea	6	15
SA/Pigeon/F4/2022	Jan. 2022	C	112	45 (40.18)	20 (17.86)	Wing paralysis, recumbency, and green diarrhea	8	20
SA/Pigeon/A1/2022	Feb. 2022	E	150	47 (31.33)	17 (11.33)	Nervous signs, head paralysis, greenish diarrhea; brain, lung, and liver congestion	10	25
SA/Pigeon/A2/2022	Oct.2022	C	70	35 (50)	10 (14.29)	Greenish diarrhea, brain congestion, and petechial hemorrhages in proventriculous	8	16
SA/Pigeon/F1/2022	Dec. 2022	G	300	88 (29.33)	33 (11)	Paralysis, green diarrhea, brain, and liver congestion	4	10

1: Swabs: tracheal and cloacal swabs.

2: Tissues: brain, proventriculous , thymus, liver ,intestine ,lung, spleen (prepared as tissue homogenates).

There were some differences in the clinical profiles and mortality rates of pigeons naturally infected with either genotype VI.2.1 or genotype VII.1.1. Pigeons infected with genotype VI.2.1 showed signs and lesions consistent with respiratory, digestive, and nervous involvements. Similar signs and lesions were observed in pigeons infected with genotype VII.1.1, except that respiratory involvement was less obvious. Both genotypes induce an almost identical overall morbidity rate of approximately 30% (156 birds out of 505 birds for genotype VI.2.1 and 178 birds out of 592 birds for genotype VII.1.1). However, genotype VI.2.1 induced a significant (*P* = 0.0009) higher overall mortality rate of 18.82% (95 birds out of 505 birds) in comparison with the 11.49% induced by genotype VII.1.1 (68 birds out of 592 birds).

### 3.4 Amino acid substitution

**Figure 5:**
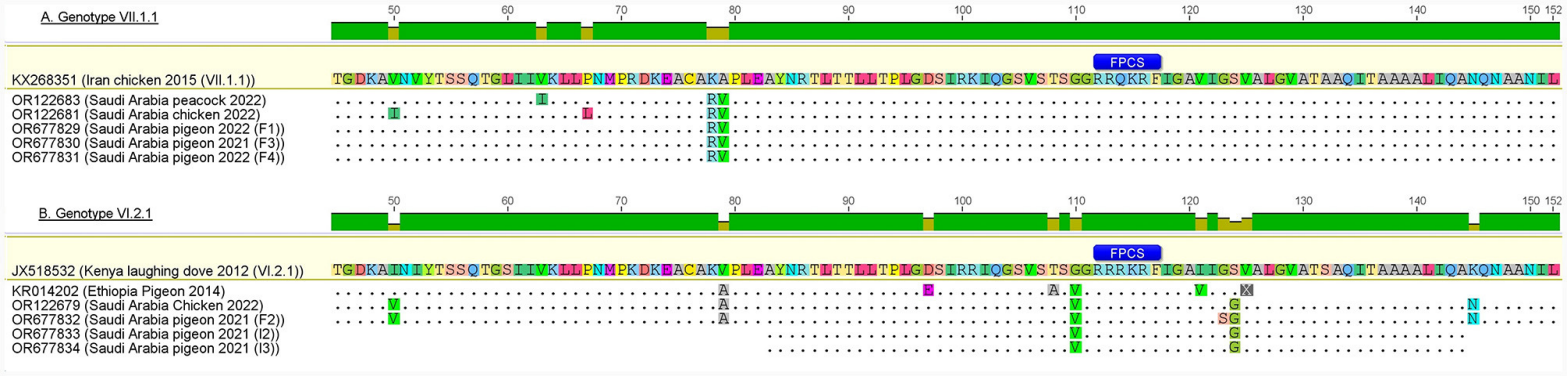
Amino acid substitutions in reported AOAV1-VI.2.1 and VII.1.1 sequences in comparison with reference and most similar sequences. **(A)** Genotype VII.1.1. **(B)** Genotype VI.2.1. The figure was prepared using Geneious 6.0.4 software (https://www.geneious.com).

Based on the deduced amino acid sequences of the FPCS (^112^RRR/QKR↓F^117^), all the reported sequences belonged to the velogenic AOAV1 strains (Table 4). The deduced amino acids for the reported sequences were compared with the most similar sequences, those recently reported from Al-Ahsa, ERSA (GB# OR122679 and OR122681). The reported VI.2.1 sequences have a single substitution (G123S), while the reported VII.1.1 sequences have two substitutions (I50V and L67P) (Figure 5). Comparison of the deduced amino acids from reported sequences with those of the most similar published sequences revealed I50V, V79A, G110V, G123S, and S124G substitutions in the reported VI.2.1 sequences and K78R and A79V substitutions in the reported VII.1.1 sequences. Of note is the tendency of the amino acid substitutions in the reported VI.2.1 sequences to locate around the FPCS.

**Table 4:** Results of testing targeted pigeons flocks.

**Sample/sequence ID**	**Real-time RT-PCR**	**Detected genotype**	**FPCS**	**GB#**
SA/Pigeon/I3/2021	AOAV1 positive	VI.2.1	RRRKRF	OR677834
SA/Pigeon/F2/2021	AOAV1 positive	VI.2.1	RRRKRF	OR677832
SA/Pigeon/F3/2021	AOAV1 positive	VII.1.1	RRQKRF	OR677830
SA/Pigeon/I2/2021	AOAV1 positive	VI.2.1	RRRKRF	OR677833
SA/Pigeon/F4/2022	AOAV1 positive	VII.1.1	RRQKRF	OR677831
SA/Pigeon/A1/2022	AOAV1 positive	ND3	ND	ND
SA/Pigeon/A2/2022	AOAV1 positive	ND	ND	ND
SA/Pigeon/F1/2022	AOAV1 positive	VII.1.1	RRQKRF	OR677829

## 4 Discussion

The poultry industry is a growing sector in Saudi Arabia, with an estimated production of 910,000 metric tons in 2021. It covered 60% of national market demand in 2020 and targets coverage of 85% by 2030. In 2021, mortalities of up to 8% of chickens were attributed to outbreaks of Newcastle disease (ND), that induced by AOAV1, and a few other viral diseases (30). AOAV1 has been known in Saudi Arabia for about four decades (19). The main goals of the current study were to investigate several outbreaks in some backyard pigeon flocks in the ERSA. It presents a detailed description of the clinical and postmortem records as well as the molecular detection and characterization of AOAV1 from naturally infected domestic pigeons in the ERSA. The phylogenetic analysis revealed the circulation of at least two AOAV1 genotypes (VI.2.1 and VII.1.1) in the investigated flocks. The present study showed the dominance of circulation of some neurotropic genotypes of AOAV1 (VI.2.1 and VII.1.1) in the investigated backyard pigeons.

Phylogenetic analysis of the reported sequences indicates that 3/6 sequences belong to genotype VII.1.1. These sequences clustered with previous detections from chickens in Iran, Pakistan, and Malaysia (32–34). Genotype VII was reported in commercial poultry in ERSA between 2012 and 2016 (22, 23). Genotype VII contains viruses responsible for the 4th ND panzootic (2) that started in the 1980s and affected Asia, Europe, Africa, and South America (35). As in many other regions of the world, viruses of genotype VII remain the leading cause of ND outbreaks in parts of the Middle East (5, 26, 36–38). Viruses of this genotype is well adapted to and maintained in chickens. It is also adapted to domestic waterfowl (35). Additionally, pigeons have been shown to be susceptible to VII.1.1 (VII d)-infection (18).

The other genotype reported in the present study, genotype VI.2.1, contains three sequences from backyard pigeons. These sequences clustered with those previously reported in backyard flocks in this region and those reported from pigeons in Ethiopia (39) in a separate cluster. Related isolates were detected in pigeons from Kenya and Nigeria in 2007–2013 (26). Similar sequences were also reported from China (GB# MN422262) and Iran (40). Genotype VI, otherwise termed Pigeon Paramyxovirus 1 (PPMV1), was reported in Saudi Arabia, the UAE, and Iran more than two decades ago (21). It was reported again in Iran in 2012 and thereafter (40). Viruses of this group are part of the 3rd ND panzootic that started in the Middle East, possibly in Iraq in 1978, and then spread to other parts of the world (3, 41). It is speculated that PPMV1 evolved from the chicken AOAV1 (3) and became adapted to columbiform (16). While circulating in the pigeon population, PPMV1 adaptation and virulence increase for pigeons and decrease for chickens (42). Beside columbiform and chickens, PPMV1 may infect many other wild birds and gallinaceous species (43, 44).

Though not detected in the present study, genotype XX was previously reported in Saudi Arabia, Iraq, Kuwait, and the UAE (21), as well as in other Asian and European countries (2). Similarly, genotypes XII, and XIII, and XXI were previously reported in the UAE and Iran (21, 40, 45) and were also reported from different countries in Asia, Europe, and Africa (2, 46).

The risk of disseminating virulent AOAV1 by wild pigeons and cormorants to poultry remains a major concern for a long time (43, 47). In the US, columbiform, especially rock doves, were involved in the maintenance of the PPMV1 (16). After infection, pigeons and cormorants may shed virulent AOAV1 for a long period, especially in faces, with no apparent clinical signs (43). PPMV1 is transmissible from infected pigeons to in-contact susceptible chickens (16, 48, 49). On infection with low-virulent PPMV1, chickens may shed the virus for up to 60 days (50). Regarding genotype VII.1.1 (VIId), it has been shown that pigeons are an efficient source of infection with this virus for in-contact chickens. Similarly, the mallard-originated VII.2 strain was shown to be readily transmissible to and between chickens (51). Recently, Hicks and colleagues conducted a phylodynamic analysis of the complete F gene sequences of AOAV1. Findings support the inter-species transmission of AOAV1-class II viruses, including those between domestic chicken and columbiform. Findings also support the intercontinental dispersion of the AOAV1-class II viruses, especially from South Asia to the Middle East (52). However, pigeons are not migratory birds and the way in which PPMV1 spreads over long distances is yet unknown. In this regard, it has been reported that closely related PPMV1 viruses were detected over the past few years from long-distant countries in Asia and Africa, including Nigeria, Egypt, Pakistan, Ukraine, Kazakhstan, and Russia (53).

PPMV1 usually shows low to moderate virulence in infected chickens (3, 16). In this regard, it has been reported that chickens near the AOAV1-VI-infected pigeons remained apparently healthy (54). After a few passages in chickens, PPMV1 eventually acquires some mutations that increase its adaptation and virulence in chickens, possibly by increasing its replication efficiency (48). However, some PPMV1 isolates were readily virulent for chickens (ICPI=1.51) with no need for prior adaptation (42). Variable ICPI scores were reported for pigeon derived PPMV1. A score mean of 1.44 and a range of 1.06 to 1.79 were reported (55). Nevertheless, lower score ranges were reported (3, 56) and wider score ranges were also reported (57). Regarding the pathogenicity of the pigeon-derived strains of the subgenotype VII.1.1, ICPI scores ranging between 1.34 and 1.96 were reported (58–60). Likewise, the previous detection of this genotype from Al-Ahsa showed ICPI score of 1.54 (22).

The present findings show that the natural infection of pigeons with AOAV1 genotype VII.1.1 induces nervus signs with greenish diarrhea and a mortality rate of 11.49%. This is in agreement with reported severe nervous signs and greenish diarrhea, along with mild respiratory signs and a mortality rate of 6.66% in pigeons after intranasal infection with the VII.1.1 virus (18). Genotype VII.1.1 strain (GB# DQ486859) replicated efficiently in the brain, cecal tonsil, trachea, and lungs of experimentally infected pigeons (61). This is also in agreement with findings showing that natural infection of pigeons with non-PPMV1 is associated with nervus signs and diarrhea (57). Nevertheless, experimental infection of pigeons with subgenotype VII.2 resulted in a 100% mortality rate. Clinical signs started with mild respiratory distress, followed by the development of nervous signs. Reported postmortem lesions include hemorrhage in proventriculus, lung, hepatic, and renal congestion (62).

The present findings showed the dominance of nervous signs and the presence of greenish diarrhea and respiratory symptoms in pigeons infected with genotype VI.2.1, with a significantly higher overall mortality rate (18.82%) than genotype VII.1.1 (11.49%). Natural infection of pigeons and doves with viruses of genotype VI.2.1 (nucleotide identity of >95%) was reported to induce Additionally, comparable nervous signs and mortality rates were observed on birds from which similar VI.2.1 sequences (> 95%) were recovered (56, 63, 64). Likewise, similar nervous signs were reported in pigeons infected with less similar isolates of genotype VI.2.1 (54, 65) and genotype VI.2.2.2.2 (66). Inconsistent clinical signs were reported after the experimental infection of pigeons with viruses of genotype VI. Pigeons infected with AOAV1-genotype VI.2.2.2.1 (GB# FJ766530) showed signs of general illness, like depression and anorexia, along with nervous symptoms and green diarrhea, with a mortality rate of 33% (67). Likewise, signs of general illness with greenish diarrhea but no mortality were also reported in pigeons infected with genotype VI.2.2.2.2 (68). On the other hand, depression and greenish diarrhea were reported in pigeons infected with strains of genotypes VI.2.1.1.2.1 and VI.2.2.2 with mortality rates of 80% and 60%, respectively (31). Nevertheless, signs of respiratory involvement in pigeons were observed following a natural infection with genotype VI.1.1 (69). Additionally, efficient viral replication in the brain, trachea, and intestine of pigeons experimentally infected with strains of genotypes VI.2.2.2.1 and VI.1.1 was reported (61, 70).

RT-PCR, followed by sequencing of the F gene, has been widely used to detect virulence and to connect AOAVs recovered from different outbreaks (65). The present study's genotyping RT-PCR failed to detect AOAV1 in samples from 2 out of the eight pigeon flocks. The false negativity of the F-based RT-PCR is well documented (71). High mutation rates and genetic variability, especially of the F gene, and the low viral RNA amount in clinical samples were reported as a possible explanations for false negative PCR results (50, 71). It has been reported that out of four validated RT-PCR assays, one was able to detect PPMV1 in all tested samples, while three assays failed to detect PPMV1 in at least one out of eight samples. PPMV1 detectability was lower in RT-PCR targeting the F gene than in those targeting the M gene. The main reason was the primer mismatching (50). A false negative result may induced by the mismatching of three nucleotides or more in a primer or probe (72, 73). On the other hand, a complete F gene sequence was recommended for the characterization of AOAV1. Dimitrov and others (2) emphasize the use of the complete F gene sequence, as well as other criteria, for the establishment of a new genotype or subgenotype. Diel and colleagues (74) propose the use of the complete F gene sequence for phylogenetic analysis. Using short F gene sequences but not the complete F gene sequences, may result in inconsistency in the topology of the phylogenetic tree (74). However, difficulties in amplifying the complete F gene of PPMV1 were previously reported (75). Additionally, Dimitrov-dataset-based phylogeny of partial F gene sequences with the right topology was previously achieved (76–82). In the present study, the topology of the obtained phylogenetic tree was maintained as that presented by Dimitrov and colleagues (2) with no out-grouping or relocation for the genotypes or subgenotypes. Additionally, each of the presented sequences aligned unambiguously and showed a similarity of >95% with reference sequences of only one subgenotype, according to the Dimitrov sequence dataset.

The deduced amino acid from reported sequences revealed the presence of polybasic amino acids (^112^RRR/QKR↓F^117^) in the FPCS, suggesting that the detected isolates are velogenic strains. Such motifs have been previously reported (83). Neutralizing epitopes at residues D72, E74, A75, K78, A79, ^157^SIAATNEAVHEVTDG^171^, and L343 were reported in the AOAV1 F protein (84–86). Two substitutions, K78R and A79V in genotype VII.1.1 and V79A in genotype VI.2.1, were reported in the present study. Similar substitutions were previously detected (GB# MH371035) in Israel and (GB# OR924274) in Bangladesh. Substitutions G123S and S124G in the fusion peptide and substitutions I50V and G110V in the F2 subunit were also observed in sequences of genotype VI.2.1 (87). Substitutions I50V and S124G were previously reported (88), as well as substitution G110V (GB#KR014202) and substitution G123S (GB#AF257759). Further research is required to identify the impact of these substitutions on the vaccine competence and virulence of the reported isolates.

AOAV1 represents a threat to domestic birds, especially commercial chickens, and wild birds, emphasizing the need to take steps to prevent the spread of the virus. Intervention may include increasing awareness among pigeon owners, implementing biosecurity measures, and vaccination. In Saudi Arabia, pigeons are not regularly vaccinated against AOAV1. Vaccination was not applied in the studied pigeon flocks, and vaccine-related strains were not detected. In contrast, vaccine-related strains were detected in commercial chicken farms (22) and wild birds, including pigeons (12). Nevertheless, viruses of genotype VI have shown some antigenic variance (2), while vaccination with genotype II strains and challenge with genotype VII.1.1 strain has shown incomplete protection (89). Inactivated PPMV1-based vaccines are recommended for pigeons to minimize their shedding for virulent AOAV1 (43). Sustainable monitoring of AOAV1 is necessary to elucidate epidemiology and take the necessary control steps. Measures to separate raised backyard species and to prevent Columbiformes from mixing or accessing water and food dedicated to poultry are also indicated to minimize inter-species transmission.

## 5 Conclusions

The present finding showed cocirculation of genotypes VI.2.1 and VII.1.1 in pigeons in Al-Ahsa, ERSA, with dominance of the neurological manifestations. It highlighted the differences in the clinical picture and mortality rates induced by AOAV1-genotypes VI.2.1 and VII.1.1 in naturally infected pigeons. These findings emphasize the need to evaluate and revise the vaccine schedules. Further research is required to understand the ecology and the different birds' roles in transmitting this virus.

## Data availability statement

The original contributions presented in the study are included in the article, further inquiries can be directed to the corresponding authors.

## Ethics statement

The animal studies were approved by King Fasial University Research Ethics Committee. The studies were conducted in accordance with the local legislation and institutional requirements. Written informed consent was obtained from the owners for the participation of their animals in this study.

## Author contributions

AA-M: Conceptualization, Data curation, Formal analysis, Funding acquisition, Investigation, Methodology, Project administration, Resources, Software, Supervision, Validation, Visualization, Writing – original draft, Writing – review & editing. AA-K: Conceptualization, Data curation, Formal analysis, Investigation, Methodology, Software, Validation, Writing – original draft, Writing – review & editing. AS: Data curation, Formal analysis, Investigation, Methodology, Software, Validation, Writing – original draft. AA: Data curation, Formal analysis, Investigation, Methodology, Validation, Writing – original draft, Conceptualization, Writing – review & editing. JH: Conceptualization, Data curation, Formal analysis, Investigation, Methodology, Validation, Writing – original draft, Writing – review & editing, Software, Supervision. MK: Conceptualization, Data curation, Formal analysis, Investigation, Methodology, Software, Supervision, Validation, Writing – original draft, Writing – review & editing, Resources, Visualization. BF: Software, Writing – review & editing. MH: Conceptualization, Data curation, Formal analysis, Investigation, Methodology, Resources, Software, Supervision, Validation, Visualization, Writing – original draft, Writing – review & editing.
