# Supplementary material for: Detection of Avian Orthoavulavirus-1 genotypes VI.2.1 and VII.1.1 with neuro-viscerotropic tropism in some backyard pigeons (Columbidae) in Eastern Saudi Arabia
Source: Front Vet Sci. 2024 Feb 27;11:1352636. doi: 10.3389/fvets.2024.1352636 (PMC10947193; doi:10.3389/fvets.2024.1352636)
Supplement: Supplementary file 1 [file Data_Sheet_1.docx]

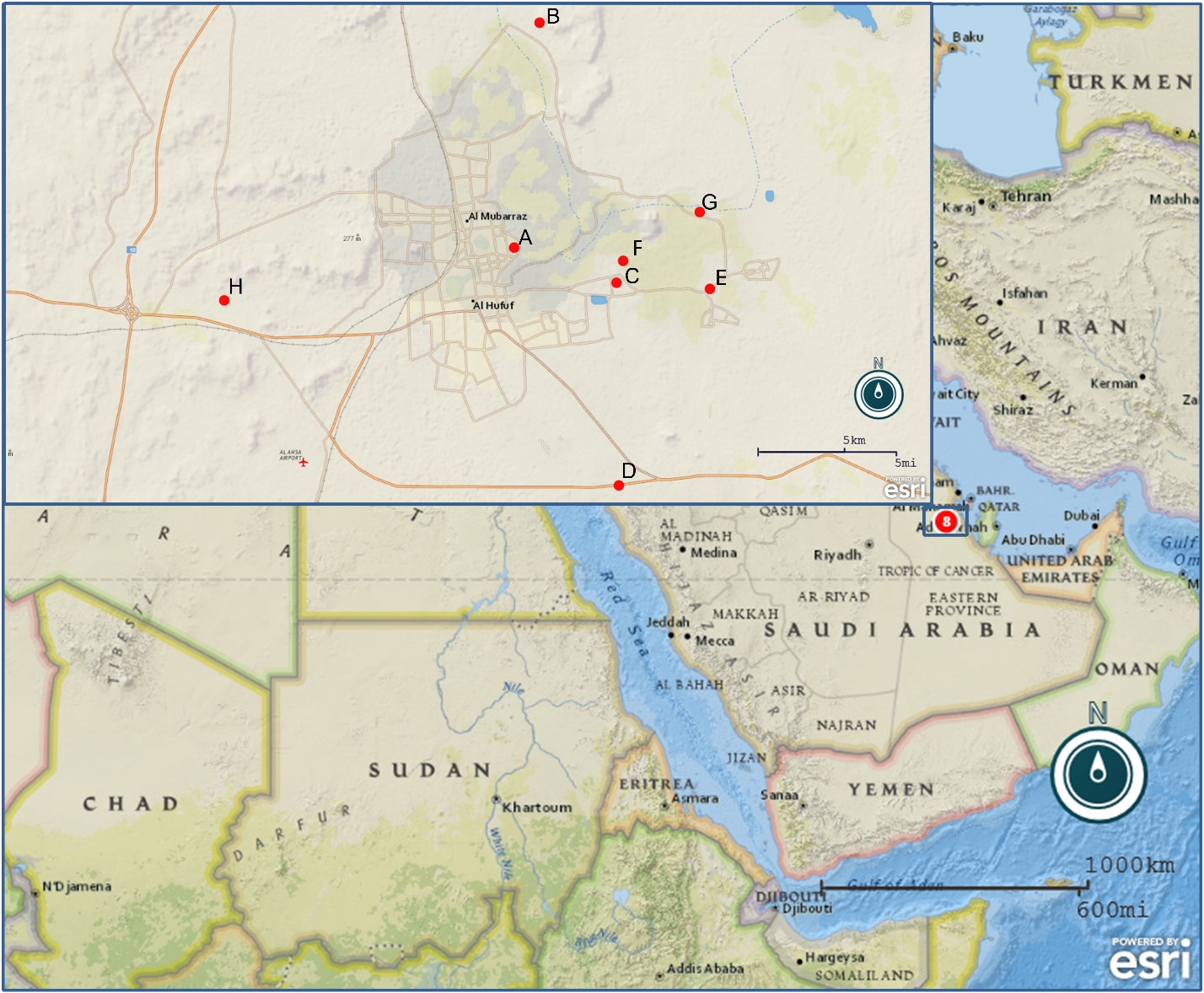


**Supplementary Figure 1:** Distribution of the AOAV1-posiive flocks in the ERSA investigated in the present study. Source of the Map: National Geographic, Esri, DeLorme, HERE, UNEP-WCMC, USGS, NASA, ESA, METI, NRCAN, GEBCO, NOAA, iPC. The map was constructed using Epi-Info^TM^ 7.2.4.0.
